# Use of Mirtazapine and Olanzapine in the Treatment of Refractory Hyperemesis Gravidarum: A Case Report and Systematic Review

**DOI:** 10.1155/2022/7324627

**Published:** 2022-09-05

**Authors:** Marco Aurélio Knippel Galletta, Vera Lucia Carvalho Tess, Isabela Marangon Pasotti, Luiza Fior Pelegrini, Nicole Kemberly Ribeiro Rocha, Carolina Burgarelli Testa, Rossana Pulcineli Vieira Francisco

**Affiliations:** ^1^Disciplina de Obstetricia, Departamento de Obstetricia e Ginecologia, Faculdade de Medicina, Universidade de São Paulo (FMUSP), São Paulo, Brazil; ^2^Instituto de Psiquiatria, Hospital das Clinicas da Faculdade de Medicina da Universidade de São Paulo, São Paulo, Brazil; ^3^Clínica Obstetrica, Hospital das Clinicas da Faculdade de Medicina da Universidade de São Paulo, São Paulo, Brazil

## Abstract

Hyperemesis gravidarum (HG) is a rare condition (1.1%) characterized by excessive vomiting, malnutrition, dehydration, and laboratorial alterations. Herein, we describe the even rarer and serious presentation of refractoriness to the usual treatment of antiemetics and parenteral nutrition, with improvement only after the use of olanzapine and mirtazapine. Two subsequent pregnancies of the same woman with HG are described, which were associated with severe weight loss, anemia, hyponatremia, hypokalemia, and mild dysfunction of liver enzymes. In the third pregnancy, the usual treatment for HG was not successful, requiring enteral nutrition and the introduction of olanzapine. In the fourth pregnancy, the patient refused to use enteral nutrition for refractory HG. Hence, the patient was started on mirtazapine at an initial dose of 15 mg/day, which was gradually increased to 30 mg/day. The patient responded well to the new regimen, as demonstrated by the decrease in symptoms, the gain of 10 kg in the pregnancy, and delivering a healthy newborn. A systematic review of literature showed 11 articles and 30 cases that successfully used mirtazapine in HG. Good clinical outcomes were seen with 4 days of the treatment and at an initial dose of 15 mg/day. However, most of these reports were from psychiatric profiles, with a predominance of depression and anxiety symptoms, and a poor description of the obstetric conditions and the disease progression itself. Pulmonary hypertension was described in one case and neonatal hyperexcitability in another. The case described in this paper reinforces the idea that mirtazapine and olanzapine can be considered in refractory HG, with good results. In the world literature, this is the second case of HG that has been successfully treated with olanzapine and the first in Latin America treated with mirtazapine.

## 1. Introduction

Hyperemesis gravidarum (HG) occurs in about 1.1% of pregnancies and seriously impacts the life quality of affected women and depletes valuable health resources [1]. It is an important cause of health expenditure and is the second leading cause for obstetric hospitalization in the United States (USA) [2].

Studies have established the loss of more than 5% of prepregnancy weight as the diagnostic criteria for HG, and its possible association with ketonuria, dehydration, hydroelectrolytic disorders, and liver or thyroid dysfunctions [3]. Mallory-Weiss lacerations, Wernicke's encephalopathy, and retinal hemorrhage are some of the maternal complications that can occur due to HG, as well as fetal congenital anomalies [4].

The treatment of HG consists of hydration, correction of hydroelectrolytic imbalance, use of antiemetics, nutritional support, hospital monitoring, and multidisciplinary care. However, the care of refractory cases remains a challenge, as the usual treatment with antiemetics fails [5]. Some unconventional therapeutic possibilities have been considered, such as antidepressants and anxiolytics. Recent reviews have identified the possibility of using mirtazapine and other psychiatric drugs as treatment for severe cases of HG, especially when associated with anxiety and depression or if refractory to usual treatments. Saks was the first to describe the successful use of mirtazapine in seven patients with HG in Tampa-Florida, USA; the patients had an associated psychiatric disorder [6]. After this pioneering article, other reports of the use of mirtazapine in HG have followed, with the ours being the first one from Latin America [7]. However, due to the rarity of the condition, reports on the efficacy of these drugs are limited.

Herein, we have described the two last pregnancies of a patient with HG refractory to the usual treatment, whose nausea reduced and appetite grew after the use of two new psychiatric medications: olanzapine and mirtazapine. By presenting this case, we aim to enlighten other physicians about the option of antipsychotic drugs for the treatment of the rare variety of refractory hyperemesis gravidarum.

To better portray the case in light of existing literature, we also carried out a systematic review of literature, looking for cases of HG treated with mirtazapine and olanzapine.

## 2. Case Presentation

We present a white woman with an obstetric score of 4G3P1A (4 pregnancies, 3 deliveries, and 1 abortion), who was in a stable relationship and has completed high school. The patient gave a family history of stomach cancer in an uncle and diabetes mellitus in a grandmother. The patient complained of nausea and vomiting in stressful situations, even before pregnancy, without other diseases or relevant personal antecedent history. She reported having HG in all four pregnancies, which progressively became more symptomatic and severe.

In her first pregnancy, during adolescence, she presented with mild nausea and vomiting, which gradually evolved to spontaneous abortion at about 5 weeks.

During her second pregnancy (in 2014) at the age of 20 years, she had intermittent nausea and vomiting throughout the pregnancy. This lead to numerous visits to the emergency department. She delivered a live, healthy female baby of 2750 g, by normal vaginal delivery.

During the third pregnancy (in 2018/2019) at the age of 24 years, she was hospitalized thrice in another hospital and eight times in ours. The first hospitalization at our hospital was at 19 weeks of pregnancy, where she presented to us with a weight loss of 10 kg (66 kg prepregnancy to current 56 kg). The total time of hospitalization at our institution was 76 days, which included two discharges on request and several readmissions after a few days, due to recurrence. Laboratory tests on admission showed anemia, hyponatremia, hypokalemia, hypocalcemia, ketonuria, and elevation of liver enzymes. She underwent digestive endoscopy, which revealed moderate erosive distal esophagitis and sliding hiatal hernia. Based on these findings, she was administered several antiemetics (metoclopramide, dimenhydrinate, and ondansetron) and gastric protectors (ranitidine, omeprazole), in addition to dexamethasone, levomepromazine, and thiamine. Despite our efforts, she kept losing weight and reached 52 kg. Due to treatment refractoriness and persistent symptoms, the use of parenteral nutrition was necessary through a central venous catheter in the right jugular vein for 5 days, in October 2018. In January 2019, due to worsening of the clinical picture, enteral nutrition was started through a nasoenteral tube, for which she was hospitalized twice, for six and four days, respectively. However, due to the persistence of lack of appetite, irritability, and insomnia, it was decided in agreement with a psychiatrist, to introduce olanzapine 2.5 mg at night. There was partial improvement of the condition, after 36-37 weeks. There was a gain of only 2 kg between the first and last hospitalizations, reaching 58 kg (body mass index of 20.8). There were no side effects, and the tolerability was good. In April 2019, she was induced and naturally delivered a live and healthy female baby of 3288-gram weight, at the gestational age of 38 weeks. The patient developed uterine hypotonia, which was reversed with uterine massage, oxytocin and ergotamine.

In her fourth pregnancy (in 2021), at the age of 26 years, she was first hospitalized at our institution at 9 weeks of gestation, after being hospitalized at another hospital, without any clinical improvement and a weight loss of 4 kg (prepregnancy 64 kg to current 60 kg), despite the regular use of two antiemetics (meclizine and ondansetron). Laboratory tests showed anemia, hyponatremia, hypokalemia, and mild dysfunction of liver enzymes. The clinical condition remained the same, despite associated therapy with metoclopramide, dimenhydrinate, bromopride, ondansetron, omeprazole, pantoprazole, and levomepromazine. We decided to introduce a central venous catheter in the right jugular vein, due to the difficulty of accessing a peripheral site. Oral diazepam of 5 mg was prescribed for night use after the second trimester of pregnancy, due to continuous insomnia, without the expected results. The patient was evaluated by a psychologist for her unhappiness related to her extended hospitalization and vomiting episodes, which had affected her quality of life and relationship with her other children. For the persistence of nausea, vomiting, lack of appetite, and weight loss, the option of enteral nutrition was considered. However, the patient refused due to the negative experience in her previous pregnancy, restricting the therapeutic options. Faced with the challenge of managing this difficult situation with the added emotional complication, insomnia, and sadness, we decided to use mirtazapine, instead of olanzapine, which has previously been used with little success. The initial dose was 15 mg at night, at 16 weeks of pregnancy, which was increased to 30 mg 24 days later, on the recommendation of the psychiatrist. Besides the tranquilizing and sleep-inducing effect of mirtazapine, an additional effect on appetite improvement was expected.

After the introduction of the mirtazapine, the patient began to gain weight again. With the highest dose of mirtazapine, there was a substantial improvement in clinical condition, with decrease in antiemetic use. The patient was discharged after four days. The patient still required three brief hospitalizations after discharge due to patient stopping the medication herself.

Till the end of pregnancy, she was hospitalized 10 times, for a total of 82 days. In the last weeks of pregnancy, the patient remained stable using mirtazapine, without major symptoms, and good tolerability. In the last hospitalization for delivery, she weighed 70 kg, with a gain of 10 kg since the beginning of the follow-up.

The patient was induced at 40 weeks and one day, in view of suspected fetal growth restriction. She delivered a female baby, weighing 2850 g, having an APGAR 9/10/10, without the need of any specialized care, by natural vaginal delivery.

At puerperal follow-up, she was in good condition and asymptomatic. Retrospectively, she agreed that the third pregnancy was the most challenging with the most intense symptoms. She admitted that the fourth pregnancy would have been even worse without the introduction of mirtazapine. After providing her with information and clarifying her doubts, the patient provided the authors with written consent for her case to be published, while respecting her right to confidentiality and not publishing her personal identification data.

## 3. Discussion and Systematic Review

Mirtazapine is an atypical, serotonergic, and specific noradrenergic antidepressant with beneficial gastrointestinal actions. It has 5-hydroxytryptamine (5HT3) antagonistic and H1 blockade actions, resulting in potent antiemesis [8]. The common side effects (≥1/10) are weight gain, increased appetite, drowsiness, and sedation. Mirtazapine is one of the few antidepressants with an affinity for H1 receptors, leading to important antihistaminergic activity. This antihistaminergic activity is the strongest predictor of weight gain with antidepressants; there is a significant association between the H1 receptor affinity of antidepressants and weight gain [9]. Thus, this medication has an antihistamine action that would have not only an important antiemetic effect but also some effect on weight gain. These characteristics have resulted in mirtazapine being used as a treatment option for hyperemesis gravidarum with relative success, as shown in the 23 case series by Spinosa et al. [5].

An additional concern with the use of mirtazapine is the teratogenic and/or psychotropic effects of this medication on newborns. However, the data so far have indicated an apparent safety in pregnancy. A recent systematic review of 31 articles with a total of 390 reported cases of exposure failed to establish an increased risk of major neonatal malformations associated with mirtazapine in pregnancy. Although one study showed an almost significant increase in the occurrence of respiratory problems and hypoglycemia, no indication of causality was given. There could be an association between mirtazapine and miscarriage; however, this could also be attributed to the underlying psychiatric illness. There is no information available on the use of mirtazapine in pregnancy and its association with neonatal maladaptation syndrome or its effect on neurobehavioral development beyond the age of one year [10].

**Figure 1:**
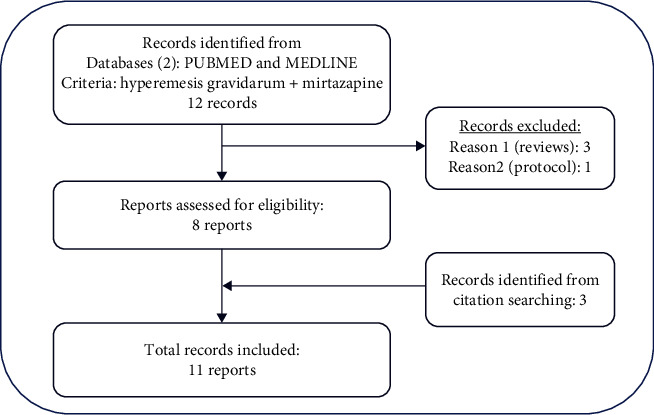
Flow chart of the selection and choice of the papers included in the review.

We ourselves carried out a systematic review on the topic. At the end of June 2022, we searched for case description articles in the PubMed and Medline databases, using the keywords, “hyperemesis gravidarum” and “mirtazapine,” that served as inclusion criteria. Cases where signs of HG were absent were discarded. Only 12 articles appeared in our search, three of which were review articles, and one was an announcement of a treatment protocol. These selected articles referred to three other case description articles, which we have also included (a total of 11 articles) (Figure 1).

Uguz et al.. describe one of the biggest case series, alongside the Saks series, that included 7 patients with major depression secondary to HG, who were admitted to the obstetrics department and then transferred to the psychiatry department [11]. Shortly before this paper, the same authors have described two cases of HG treated with mirtazapine, although it is unclear if these patients are the same as the ones in the case series [12]. In general, these patients had a gestational age 7–14 weeks (mean 11.57 weeks) at the beginning of the treatment. The duration of treatment ranged 4–24 weeks (mean 9.14 weeks), with medication dose ranging 7.5–15 mg per day. Only one patient had a recurrence of depressive symptoms, nausea, and vomiting, which subsided on mirtazapine reintroduction. None of the patients were hospitalized for symptoms of HG after mirtazapine treatment. The studies do not mention the gain or loss of weight or the obstetric results.

In the eleven selected articles, a total of 28 reported cases of HG treated with mirtazapine were reported (Table 1). If the two cases by Uguz et al. were considered to be unpublished, there would be 30 cases [12], and none of them in Latin America. These case reports have been described in Europe, North America, and Turkey [6, 11–20]. After analysis of each patient, we have a global assessment of data, as shown in Tables 2 and 3.

Most of the cases in the papers were described by psychiatrists; therefore, obstetric data is limited. There is information on the newborn weight in only 8 of the case reports, the type of delivery in 13, and the gestational age at delivery in 16 cases. The newborn's sex was included in only 11 cases, showing a surprising predominance of males (9/11 = 81.8%). A description of weight loss is mentioned in only 11 cases and laboratory alterations or dehydration in 3 cases. The need of parenteral nutrition was described in 7 cases (Table 2). Most of the papers describe only the presence of nausea and vomiting, and a diagnosis of hyperemesis gravidarum is unclear. The lack of such details makes us wonder if the high effectiveness demonstrated would be the same in severe, well-defined cases of HG.

Our case report is more complete, with consistent data on obstetric follow-up and the birth itself. Our patient was younger and with greater parity than the average of the other cases and had a female newborn, in contrast to the predominance of male babies in the studies. The initial weight loss was comparatively less (4 × 7 kg) than that described in other studies. The initial dose of mirtazapine, 15 mg per day, was similar to that in other studies. However, the dose was not increased to 30 mg in all reports. The effect was equally fast and beneficial as the other reports, with the patient gaining almost 10 kg during pregnancy. Only two authors reported weight gain in their patients: 2.5 kg in Arshad et al. and almost 16 kg for one of the cases described by Saks [6, 20]. The number of antiemetic medication used was higher than the average of the other papers (8 × 2.8) and higher than the maximum used in other case reports [8]. Our patient had a more severe condition with several laboratory alterations, which was described in only 3 cases. The newborn's weight (2850 g) was similar to the average of 2644 g in the other reports (Table 3). Our patient was hospitalized several times, with 3 hospitalizations after the introduction of mirtazapine, which may be associated with the low home adherence, comparable to findings in the other studies. The newborn did not have neonatal complications, which did occur in 21 of the 23 complete reports. One newborn was described by Saks, where the newborn was diagnosed with pulmonary hypertension who needed ventilator support in the neonatal intensive care unit for 3 days [6]. The newborn described by Schwarzer et al.. developed hyperexcitability, tremors, tachycardia, and tachypnea, which spontaneously ceased after 36 hours [16]. The delivery in this case was anticipated at 34 weeks due to preeclampsia and fetal growth restriction, and the dose of mirtazapine used was one of the highest, 45 mg per day.

The use of mirtazapine proved to be equally beneficial in our case and in the other cases described in literature. This indicates that mirtazapine is a good treatment option for hyperemesis gravidarum, when conventional treatment fails. Furthermore, it would be useful prior to initiation of invasive modes, like enteral or parenteral nutrition, or in cases where the patient refuses treatment, like in major depressive episodes.

The use of olanzapine in our patient's third pregnancy had good outcomes but with a lesser control of the symptoms, making us switch to mirtazapine in the subsequent pregnancy.

There is a lack of data in literature on the use of olanzapine in HG. When reviewing the use of olanzapine in two databases, PubMed and Medline, using the keywords, “hyperemesis gravidarum” and “olanzapine,” we found only two articles. One of them was a description of HG that was satisfactorily treated with olanzapine, in a 26-year-old woman with relapsed depression. She was 14 weeks pregnant, was dehydrated, and had lost more than 9 kg, when she was started on 5 mg/day of olanzapine. She improved within 5 days [21]. Olanzapine was successfully used in a case of cannabinoid hyperemesis, a condition characterized by nausea, vomiting, and abdominal pain in marijuana users [22]. Little is known about the safety of the use of this medicine in pregnancy, even though some recent reviews have established apparent safety [23, 24].

Our study has some strengths and limitations. The present report is not the first or the only one that describes the use of mirtazapine in HG, and there is a lack of some details. But it is the first to be described in Brazil and Latin America. The disease is infrequent and can be extremely serious, especially if refractory to the traditional antiemetics. However, there is a lack of obstetric data in studies with mirtazapine use. A strength of our report is the fact that it has a more detailed description of the obstetric data and a complete review of such aspects in other reports. This provides a better idea of what type of patient would benefit from mirtazapine use. Additionally, we also presented the use of olanzapine in HG, which may be a viable option in some cases.

The reported case reinforces the fact that some psychiatric medications, such as olanzapine and mirtazapine, have side effects such as increasing appetite, while also reducing nausea, which is beneficial in HG.

Based on our personal experience with such cases, we saw that “having nothing else to do” in a situation of prolonged suffering often leads to severe emotional distress in the patients, including depression and desire for abortion. More such studies are required, due to the rarity of the condition, before these medications can be established for routine use. Our experience in this case showed us that these medications are especially beneficial when there is underlying depression or anxiety.

## 4. Conclusion

This case is the first to be described in Latin America that used olanzapine and mirtazapine in refractory HG. Our paper reinforces the beneficial effect of these medications on gastrointestinal symptoms. They improve the nutritional status, cause weight gain, and reduce the need for invasive procedures. Furthermore, it prolongs pregnancy allowing fetal weight gain. Although obstetric outcomes were similar with olanzapine and mirtazapine, mirtazapine had better symptomatic control. Mirtazapine plays an important therapeutic role in refractory HG, without any harmful repercussions to fetal development. Thus, it can be used to significantly improve the maternal and fetal outcomes in HG.

## Figures and Tables

**Table 1:** Publications about the use of mirtazapine as treatment for HG with some details. The first ten provided a detailed description of each patient and provided data for the analysis of the following tables. The last publication was only a summary of the cases, without details.

Author	Place	Medical field/specialty	No. cases
Saks, [6]	Tampa, Florida, USA	Psychiatry	7
Dorn et al., [14]	Bonn, Germany	Obstetrics and gynecology + psychiatry	1
Rhode et al., [15]	Bonn, Germany	Obstetrics and gynecology + pediatrics + psychology	1
Guclu et al., [19]	Ismir, Turkey	Obstetrics and gynecology	3
Scharzer et al., [16]	Bonn, Germany	Obstetrics and gynecology + pediatrics + psychology	1
Lieb et al., [17]	München, Germany	Psychiatry	1
Uguz, [12]	Konya, Turkey	Psychiatry	2
Arshad et al., [20]	Doncaster, United Kingdom (UK)	Gastroenterology + endocrinology + obstetrics and gynecology	1
Omay and Einarson, [18]	Tain l'Hermitage, France and Ontario, Canada	Psychiatry + pharmacology	5
Spiegel et al., [13]	Norfolk, Virginia, EUA	Psychiatry	1
Uguz et al., [11]	Konya, Turkey	Psychiatry	7

**Table 2:** Data from 23 case reports of pregnant women with hyperemesis gravidarum (HG) treated with Mirtazapine. Maternal disease: two patients with type 1 diabetes and one patient with lyme disease. Obstetric pathology: gestational diabetes, preeclampsia, twinning, abnormal Doppler velocimetry, and multiple fetal malformations (trisomy 18). Neonatal complications: hyperexcitability and pulmonary hypertension.

Variable	*N*	Yes	Total Percentage	Valid Percentage
Primiparous	21	6	26.1%	28.6%
Nulliparous	21	9	39.1%	42.8%
Previous HG	19	7	36.8%	30.4%
Previous interruption of pregnancy due to HG	10	5	21.7%	50%
Desire of interruption of current pregnancy	11	7	30.4%	63.3%
Depression	14	13	56.5%	92.8%
Anxiety	11	10	43.5%	90.1%
Weight loss	11	11	47.8%	100%
Laboratorial alteration	3	3	13%	100%
Dehydration	3	3	13%	100%
Parenteral nutrition	12	7	30.4%	58.3%
Use of intravenous mirtazapine	21	5	21.7%	23.8%
Clinical improvement with mirtazapine	23	22	95.7%	95.7%
Use of ondansetron	16	9	39.1%	56.3%
Maternal disease	23	3	13%	13%
Obstetric pathology	23	5	21.7%	21.7%
Cesarean delivery	13	8	34.8%	61.5%
Male newborn	11	9	39.1%	81.8%
Neonatal complication	13	2	8.7%	15.4%

**Table 3:** Numerical data of the 23 cases described in the literature of pregnant women with hyperemesis gravidarum treated with mirtazapine.

	*N*		Mean	Median	Minimum	Maximum	Percentiles		
	Valid	Omit					25	50	75
Age (years)	22	1	30.45	31.00	20	38	28	31	34
Gestational age (weeks)	23	0	12.30	11.00	5	25	9.00	11.00	15.00
Gesta	21	2	2.48	2.00	1	6	1.0	2.0	3.5
Para	21	2	0.71	1.00	0	2	0	1.0	1.0
Abortion	21	2	0.76	0	0	4	0	0	1
Weight loss (kg)	8	15	7.60	7.00	2.5	13.0	3.500	7.000	11.825
Initial dose (mg/day)	23	0	15.3587	15	3.75	30	7.5	15	15
Effective dose (mg/day)	22	1	20.2841	15	3.75	45	15	15	30
Days for clinical improvement (days)	14	9	4.14	3	1	14	2.00	3.00	4.25
Treatment time (days)	12	11	56.50	21	2	210	6.75	21	119
Number of antiemetics	16	7	2.88	3.0	1	6	2	3	4
Gestational age at birth (weeks)	15	8	37.07	37	30	40	35	37	40
Newborn weight (grams)	8	15	2644.63	3204.50	1102	3550	1647.50	3204.50	3329.50

## Data Availability

Additional details about the case in question or the literature review may be provided upon reasonable request to the corresponding author.
